# Additive Manufacturing of Spinal Braces: Evaluation of Production Process and Postural Stability in Patients with Scoliosis

**DOI:** 10.3390/ma15186221

**Published:** 2022-09-07

**Authors:** Fabio Alexander Storm, Davide Felice Redaelli, Emilia Biffi, Gianluigi Reni, Paolo Fraschini

**Affiliations:** Scientific Institute IRCCS “E. Medea”, 23842 Bosisio Parini, Italy

**Keywords:** additive manufacturing, scoliosis, spinal orthoses, postural stability, production process

## Abstract

Spinal orthoses produced using additive manufacturing show great potential for obtaining patient-specific solutions in clinical applications, reducing manual operations, time consumption, and material waste. This study was conducted to evaluate the production process of spinal orthoses produced by additive manufacturing, and to test the effects of 3D-printed braces on postural stability in patients with adolescent idiopathic scoliosis and osteogenesis imperfecta. Ten patients were recruited consecutively and were asked to wear a spinal orthosis produced by additive manufacturing for 2 weeks. The four phases of the production process for each brace were evaluated separately on a scale from 0 (not acceptable) to 3 (optimal). Postural stability in the unbraced and the two braced conditions (3D-printed and conventional) was assessed using validated metrics obtained from a wearable inertial sensor. The production process was evaluated as good in four cases, acceptable in five cases, and not acceptable in one case, due to problems in the printing phase. No statistically significant differences were observed in any of the postural balance metrics between the 3D-printed and conventional brace. On the other hand, postural balance metrics improved significantly with both types of braces with respect to the unbraced condition. Spinal orthoses produced with an innovative production process based on digital scans, CAD, and 3D printing are valid alternatives to conventionally produced orthoses, providing equivalent postural stability.

## 1. Introduction

Spinal orthoses, also known as braces, are orthotic devices used for the correction and prevention of spinal deformities. They are a well-documented form of treatment for controlling the progression of scoliotic curves, and an essential part of the larger process of rehabilitation. Scoliosis is a structural abnormal curvature of the spine in the coronal plane, and is commonly diagnosed using standard posteroanterior radiographs [1,2,3]. Two typical conditions where spinal orthoses are recommended are adolescent idiopathic scoliosis (AIS) and osteogenesis imperfecta (OI) [4,5]. AIS is the most common type of scoliosis, affecting 1–4% of adolescents, and denotes a curve of unknown aetiology [6]. OI identifies a group of inherited bone dysplasias characterized by bone deformities caused by bone fragility and low density, where scoliosis is a typical secondary feature [7].

Prefabricated orthoses can be uncomfortable and have poor fit, causing pain and problems of pressure and perspiration, potentially causing functional loss and reducing compliance [8]. Prefabricated orthoses can be replaced with thermoplastic orthoses customized for a given patient, leading to improved results overall [9].

**Figure 1:**
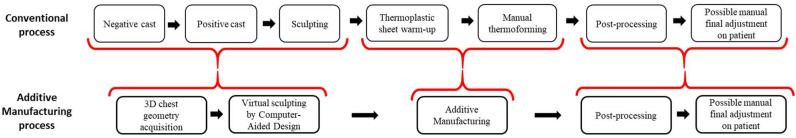
Conventional and additive manufacturing processes for braces.

As shown in Figure 1, the conventional process currently used by most orthotists to produce customized spinal braces is based on a positive mold made by plaster casting or through 3D scanning, computerized milling, and thermoforming. This process is time-consuming, done mostly manually, and not environmentally friendly [10,11]. Recent technological advances include employing computer-aided design/computer-aided manufacturing (CAD/CAM) and 3D scanning to generate a virtual geometry of the patient as an alternative to manual casting and rectification [12,13].

Additive manufacturing, commonly known as 3D printing, is a technique that creates objects from 3D digital models, showing great potential for obtaining patient-specific solutions in clinical applications by replacing the traditional thermoforming phase used in orthosis and prosthesis (O&P) production [14,15] and spine surgery [16]. Compared with the traditional manufacturing of custom-made orthoses, this method may reduce skill-based manual operations, time consumption, and material waste [17,18]. Further advantages of customization include improved accommodation of inter-individual anatomy variations [19] and cost-effectiveness. Additive manufacturing based on fused deposition modeling (FDM) techniques consists of producing 3D objects layer-by-layer using thermoplastic polymers. A few papers proposed the use of FDM to fabricate orthotic devices, mainly foot and wrist orthoses. Several materials have been experimentally tested with FDM, including acrylonitrile butadiene styrene (ABS) for foot and wrist orthoses and casts [20,21,22], thermoplastic polyurethane (TPU) for insoles [23], polylactic acid (PLA) and thermoplastic polyester (TPE) for prosthetic sockets [11], and nylon-12 for braces [24].

Only very recently, a randomised clinical trial evaluated the clinical effectiveness of orthoses produced by FDM in the management of AIS [25,26]. The results showed comparable clinical effectiveness, compliance, and quality of life between patients treated with 3D-printed orthoses and the control group. In addition, 3D-printed orthoses were thinner and lighter when compared with conventional orthoses produced for the same patients. Finally, the orthotist saved 4.8 person-hours in the design and fabrication of the 3D-printed version of the orthoses. Previous research has focused on specific aspects of the production process, such as mold rectification [27] and semi-automatic design systems for generating printable models [28]. However, very limited information is available related to the evaluation and critical appraisal of the separate phases of the additive manufacturing process used to fabricate 3D-printed orthoses.

The influence of bracing on postural stability is still controversial; some research has shown that bracing does not influence standing balance in AIS [29,30]. However, others report an improvement in postural stability of patients wearing a Chêneau brace [31]. A literature review also showed that the use of orthoses influences the symmetry of gait and can improve the standing stability of patients with scoliosis [32]. Experimental research has shown that patients with scoliosis due to AIS and OI show greater postural instability compared with age-matched controls [33,34]. Bracing may have an influence on standing balance, because of its influence on trunk morphology and curve types [35,36]. Postural stability is traditionally investigated using force platforms, capable of measuring parameters associated to excursion and sway of the centre of pressure. However, body sway may also be investigated by means of body-worn accelerometers, a low-cost alternative based on sensors usually positioned at the lower back in order to measure the dynamics of the centre of mass [37]. A direct comparison of the effects on posture and body sway between a 3D-printed and conventional spinal orthosis in patients with AIS and OI using body-worn sensors has not yet been performed. Building on previously published work that focused on existing production processes [10], material selection [38], validation of scanners for accurate 3D geometry [39], and complete characterization of printed polymers in terms of mechanical, morphological, rheological and thermal characteristics [40], the objectives of the paper are twofold:

1. To quantitatively evaluate all phases of an innovative production process to produce spinal orthoses based on FDM additive manufacturing for the treatment of scoliosis in AIS and OI patients.
2. To compare postural stability metrics between the unbraced and the two braced conditions (3D-printed and conventional), obtained using a wearable inertial sensor.

We performed a pilot study where patients were asked to wear a spinal orthosis produced by additive manufacturing for 2 weeks. The phases of the production process were evaluated on a scale from 0 (not acceptable) to 3 (optimal). Postural stability was assessed using validated metrics obtained from a wearable inertial sensor. The production process was evaluated as good or acceptable in nine cases, and not acceptable in one case. No statistically significant differences were observed in any of the postural balance metrics between the 3D-printed and conventional brace. Our preliminary results suggest that spinal orthoses produced with an innovative production process based on digital scans, CAD, and 3D printing are valid alternatives to conventionally produced orthoses, providing equivalent postural stability.

## 2. Materials and Methods

### 2.1. Study Design and Participants

The investigation was designed as a single-group pilot study and was performed in accordance with the Declaration of Helsinki. The Ethics Committee of the Scientific Institute E. Medea approved the study protocol. Written informed consent was obtained from patient parents. The study protocol was registered in Clinicaltrials.gov (NCT04282408). Participants were recruited from the outpatient clinic based on inclusion and exclusion criteria. Inclusion criteria for patients with osteogenesis imperfecta included the following: between 3 and 17 years of age; experiencing vertebral pain and/or vertebral deformity with double curve shape and/or deformity in frontal or sagittal planes assessed with clinical traction; and conventional brace users for at least 1 year before recruitment. Inclusion criteria for patients with AIS included the following: between 6 and 17 years of age; diagnosed with lumbar or thoracolumbar scoliosis; and conventional brace users for at least 1 year before recruitment. Exclusion criteria for all patients included: presence of skin allergies; behavioural problems; and chest dimensions >35 cm in diameter or >60 cm in height.

At Study Visit 1, all patients completed an instrumented postural stability test in both unbraced and thermoformed braced conditions. Then, a chest geometry scan was performed for each patient. At Study Visit 2, usually 2 weeks after Visit 1, the patient received the 3D-printed brace and started to wear it continuously for 2 weeks, according to the previous orthotic treatment prescription. The brace was worn above a light t-shirt to improve comfort and compliance, as well as reducing the risk of skin irritation. During this period, the patient completed a diary reporting the wearing hours of the brace. At Study Visit 3, after 2 weeks of continuous wearing of the 3D-printed brace, we tested patient postural stability again in both unbraced and 3D-printed braced conditions. After the 2 weeks of testing, patients returned to wearing their conventional brace.

### 2.2. Production of the 3D-Printed Braces

The production process of the 3D-printed braces consists of 4 phases and is suitable for real-world applications in O&P workshops, because it does not rely on the existence of a conventional orthosis, necessary in the case of reverse engineering techniques used in our previous case study [38].

#### 2.2.1. 3D Chest Geometry Acquisition

An infrared triangulation scanner (Structure Sensor, Occipital Inc., Boulder, CO, USA) with adequate accuracy for O&P applications [39] was used to obtain 3D chest geometry. The device was connected to a tablet (iPad Pro, Apple Inc., Cupertino, CA, USA) and controlled via software (Scanner, Occipital Inc., Boulder, CO, USA). The bounding box of the scanner was limited superiorly by the armpit, and inferiorly by the gluteus.

#### 2.2.2. CAD Design Process

**Figure 2:**
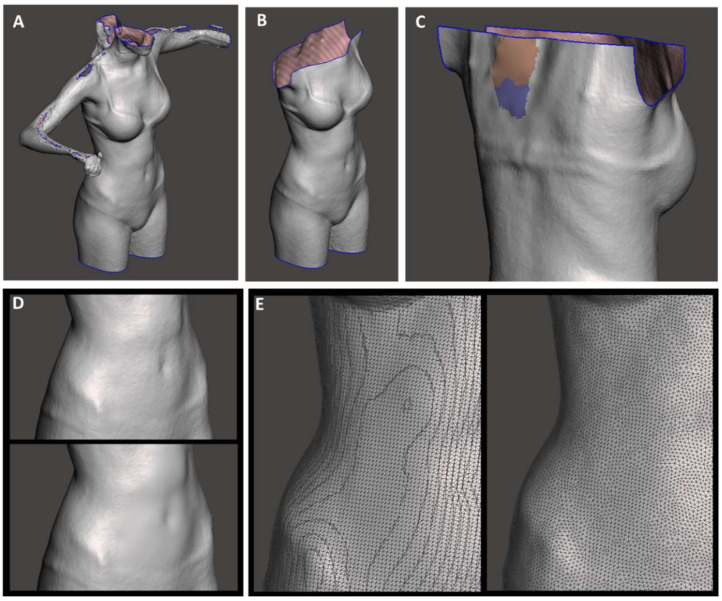
Preprocessing phases: (**A**) raw scan; (**B**) removal of unnecessary body parts; (**C**) closing holes in the mesh; (**D**) local smoothing without re-mesh; (**E**) zoom on the right side before (left) and after (right) global re-mesh.

The general-purpose sculpting CAD software MeshMixer v.3.3.15 (Autodesk, San Rafael, CA, USA) was used to verify the mesh quality of the scanned 3D models, to remove artefacts, repair the mesh, and smooth the surface. A final re-mesh was performed to obtain a uniform mesh geometry (Figure 2). 

**Figure 3:**
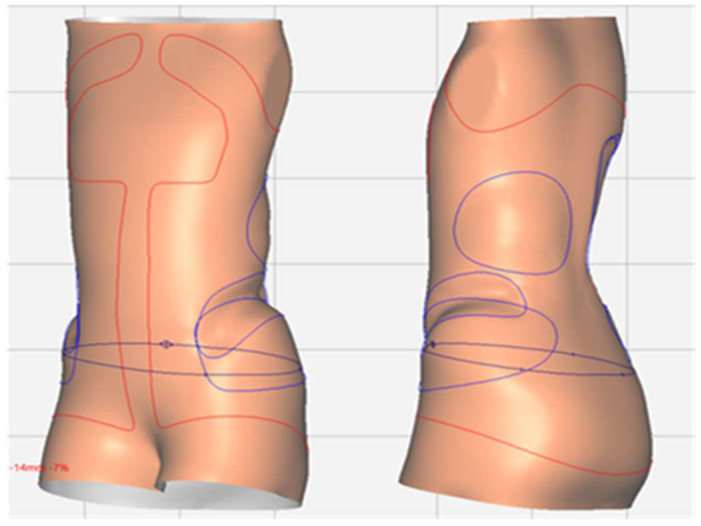
CAD design using a specialized software for O&P products (Neo, Rodin4D, Merignac, France).

A specialized software for the design of O&P products (Neo, Rodin4D, Merignac, France) was used for the specific operations necessary for the creation of the brace model: vertical stretching, flexion, sculpting, expansions, smoothing, cutting, and exporting as an open surface (Figure 3). 

**Figure 4:**
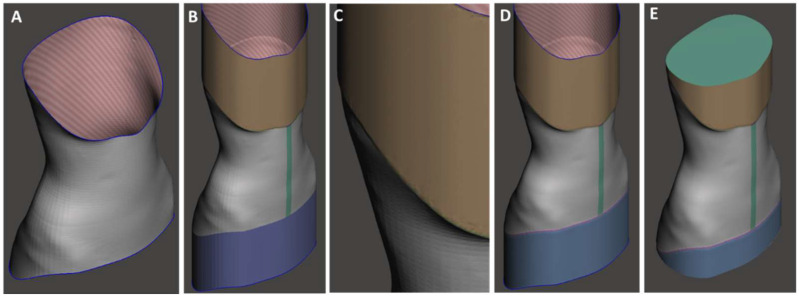
Final steps of the CAD modelling phase: (**A**) CAD model after modelling phase with specialized O&P software; (**B**) vertical extrusion of the borders; (**C**) detail of the offset on the extruded part; (**D**) re-mesh and smoothing; (**E**) plane cut and surface closure.

A vertical extrusion of the borders was then performed using MeshMixer, serving inferiorly for support and better adhesion to the build plate and superiorly to improve the printing quality of the final layers, avoiding nozzle retractions that could cause stringing. Finally, an offset was performed on this surface to create an external curvature to the brace border and avoid sharp edges (Figure 4).

#### 2.2.3. Additive Manufacturing 

**Figure 5:**
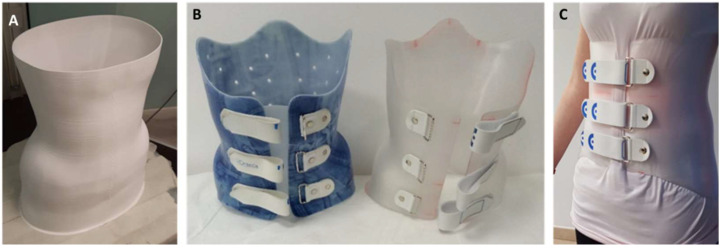
Three-dimensional printed orthosis production: (**A**) orthosis on the build plate before post-processing, (**B**) 3D-printed orthosis (right) compared with thermoformed orthosis (left), and (**C**) 3D-printed orthosis worn by the patient.

The g-code files that control the 3D printer were prepared with a slicing software (Cura, Ultimaker, Utrecht, The Netherlands). The thickness of the braces was set to 2.2 mm. Typical printing parameters are shown in Table 1. The brace was printed using an FDM printer (Delta 4070 Pro, Wasp, Massa Lombarda, Italy), equipped with a 1.2 mm nozzle. A commercial 1.75 mm diameter polyethylene terephthalate glycol-modified (PETG) filament (Zhuhai Sunlu Industrial Co., Zhuhai, China) was used as output material (Figure 5A). PETG is a thermoplastic copolyester, commonly used in medical applications due to its high transparency, heat resistance, workability, low toxicity, low gas permeability, and ease of sterilization. Tensile tests performed on FDM specimens of PETG, PLA, and conventional thermoformed specimens of polypropylene (PP) and polyethylene (PE) [38] showed that, as expected, FDM specimens presented different mechanical responses depending on fiber direction. However, when the load was applied longitudinally to the fibers, the behavior was comparable to that of PP and PE, thanks to the cross-sectional resistance of the fibers. Overall, PETG samples revealed mechanical properties similar to PP samples in terms of elastic modulus and ultimate tensile strength. PE samples performed better in terms of elongation at break but resulted in less stiffness than PETG samples. Further mechanical characterization [40] studying the effects of material and print orientation on the modulus and strength of the printed samples showed that 3D-printed PLA-based samples have inferior mechanical proprieties when compared with PETG samples. Overall, PETG appeared to have the appropriate mechanical properties for the present application. 

The layer height can usually reach up to 75% of the nozzle diameter. In our case, we found the best trade-off between print quality and printing speed was around 35–50%. The width of the extruded line is generally equal to the nozzle diameter but can be varied to ±10% in combination with the material flow. In our case, using the 1.2 mm nozzle, two 1.1 mm lines allowed us to reduce the line spacing and thickness of the brace (2.2 mm overall). The value of the line width was increased by 10% only for the first layer to allow better adhesion to the print bed. The Z-seam alignment was positioned close to the midline of the brace, where the opening would be cut. Printing temperature and build plate temperature were set according to filament producer specifications. Flow was set at 103% to obtain a good supply of material and reduce the possible void between the 1.1 mm lines.

#### 2.2.4. Post-Processing

The brace was removed from the 3D printer build plate, and by-products (i.e., brim layers and additional extruded ends used as supports) were removed manually or using pliers. An experienced orthotist completed the finishing of the brace by smoothing borders and surfaces and applying closure straps, using a grinder, drill, and riveter (Figure 5B,C).

### 2.3. Production of the Conventional Braces

The conventional thermoformed braces were produced using a traditional fabrication process involving 3D chest geometry acquisition by 3D scanning, CAD design for the creation of the brace model using a specialized software (Neo, Rodin4D, Merignac, France), a computer numerical control (CNC) milling machine for the production of a positive mold made of expanded polyurethane, heating and vacuum-forming sheets of thermoplastic (commonly polyethylene) onto the positive mold, and final trimming.

### 2.4. Analysis of the Production Process

The phases of the production process of each 3D-printed brace were evaluated individually, on a scale from 0 (not acceptable) to 3 (optimal), according to the criteria presented below (Table 2). The criteria were defined by consensus among a multidisciplinary group composed of engineers, orthotists, and physicians. In addition, all adverse events were recorded and monitored.

### 2.5. Postural Stability

Postural stability was assessed using a wireless wearable device with an embedded tri-axial accelerometer (G-Sensor, BTS, Milano, Italy) positioned on the lower back at L5. The raw output data of the sensor was sampled at 100 Hz. Data was transmitted to a laptop using Bluetooth, and processed offline in MATLAB (R2020b, MathWorks, Natick, MA, USA). Data was collected for each condition (3D-printed brace, conventional brace, unbraced) for 60 s with patients in a standing posture, eyes open and both feet together. Balance metrics shown in Table 3 were computed using validated algorithms [41,42,43]. Friedman tests with Dunn-Bonferroni post hoc tests were used to establish whether the parameters varied among the three testing conditions (*p* < 0.05).

## 3. Results

### 3.1. Patients

Ten patients were consecutively enrolled in the pilot study, eight with AIS (eight women, aged 12.8–17.3 years) and two with OI (two men, aged 6.9–8.5 years). Median (25–75th percentile) values for height, weight, BMI, and Cobb angles were 162 cm (158–167 cm), 42 kg (40–47 kg), 16.9 kg/m^2^ (15.6–17.4 kg), and 31° (24–36°), respectively. Overall, wearing time was 9.9 h/day (6.9–11.8 h/day).

### 3.2. Analysis of the Production Process

Table 4 summarises the results of the production process evaluation: seven scans were considered optimal, two cases were considered good, and one case was classified as acceptable because the scans had to be repeated due to the inability of the patient to stand quietly during acquisition. The CAD design process lasted from a minimum of 40 min to a maximum of 2 h, with seven cases classified as good, one as optimal, and one as acceptable. Only one case was classified as not acceptable due to failed 3D printing. For the additive manufacturing phase, the first attempt was enough for eight cases, a second attempt was needed for one case, and one case required five attempts. Finally, the duration of the post-processing phase ranged between 40 min and 1 h 40 min, with six cases classified as good and four cases as acceptable. The manufacturing was completed according to plans and only the first attempt ended with a failure of the print, due to frequent retractions of the nozzle that caused air bubbles, making the brace prone to cracking and breaking. The retractions were removed by changing the print setting with wall thickness equal to 2.2 mm, which allowed production of the whole brace with only two wall lines.

There were two non-serious unexpected adverse events: a small crack in the brace worn by patient ID 3, which occurred while resting on a hard surface and a crack under the armpit of the brace worn by patient ID 9, which occurred while walking.

### 3.3. Postural Stability

**Figure 6:**
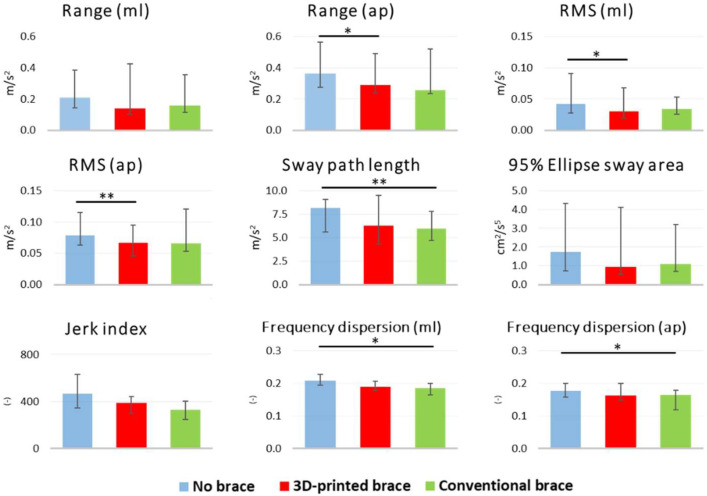
Postural stability results. Significance codes: ‘**’ 0.01; ‘*’ 0.05.

No statistically significant differences were observed in any of the postural balance metrics between the 3D-printed and conventional brace. On the contrary, the anterior-posterior acceleration range (AP range) and root mean square (RMS) were significantly lower in the 3D-printed braced condition compared with the unbraced condition. In addition, sway path length and frequency dispersion values were significantly lower in the conventional braced condition compared with the unbraced condition (Figure 6). 

## 4. Discussion

The main goal of the present study was to assess an innovative production process for spinal braces produced by additive manufacturing, separately evaluating all production phases. In addition, we also evaluated acceptance and postural stability in a group of patients with AIS and OI.

The production process presented and tested in this pilot study, based on digital scans, CAD, and 3D printing, is an innovative method that eliminates the need for plaster casts and manual thermoforming of the brace. Compared with conventional orthosis production, this method eliminates the need for expensive equipment, including CNC milling machines and thermoforming ovens.

In a recent clinical trial, 3D-printed orthoses were produced and tested [25,26], showing comparable clinical effectiveness to conventional orthoses. In the present pilot study, we performed a detailed analysis of the different phases of the production process of a 3D-printed spinal orthosis on ten patients affected by AIS and OI, separately evaluating chest geometry acquisition, 3D CAD design, additive manufacturing, and post-processing. Such a detailed evaluation has not been previously reported. 

Only the production of the first orthosis resulted in repeated attempts due to failed 3D printing and consequent troubleshooting of the design and printing parameters. Similar to a previously reported prosthetic socket production method based on FDM [44], subsequent attempts resulted in the reduction of fabrication time, with all braces produced within one working day, including the 3D-printing phase, with no full-time involvement of an engineer or technician. This confirms the findings of a previous case study preliminarily demonstrating the feasibility and cost-effectiveness of spinal braces produced by FDM [38].

The overall process was considered to be satisfying and competitive by both physicians and orthotists, with improvement in the process and reduced handwork, as reported by [25]. 

### 4.1. 3D Scanning

For 3D scanning, we used a handheld device experimentally validated against a triangulation-based laser scanner [39]. Its correct usage depends on the smooth motions made by the operator along the scan path [20]. This setup allowed for the correct acquisition of chest geometry for most of the patients. However, one patient had difficulty in standing still for 20–30 s, resulting in low-quality scans that needed to be repeated. Several full-body scanners based on structured light are available on the market and may overcome this issue; however, full-body scanners may be prone to disadvantages such as potential occlusions, high costs, and a large space required for installation.

### 4.2. CAD Design

For several reasons, CAD modelling of a 3D chest geometry to produce braces through an additive manufacturing process requires more time compared with modelling a conventional CAD file that is fed to a CNC milling machine to make a positive mold. The limited opportunities of the additive manufacturing process for making manual adjustments results in the need for higher precision in the geometry of the model. A border expansion on the CAD model is also required to highlight the brace border, which requires additional time. The CAD phase was optimized throughout the study: in the first attempt, the 2 mm thickness of the brace was set during the CAD modelling phase, with a perpendicular expansion from the model surface. Later, this operation was performed in the slicing software using the horizontal expansion option, which proved to be more effective in ensuring a constant horizontal thickness throughout the printed brace, avoiding filament retraction during printing. Improvements in this phase may come from creating a standardized workflow within a single software suitable for any required operation, as suggested by other pilot studies [11,28].

### 4.3. Additive Manufacturing

Besides the first attempt ending with the failure of a print, the improvements in the CAD design described previously allowed for the realization of all remaining braces on the first attempt. Future improvements may include manufacturing the brace directly with frontal openings and no vertical extrusions, to reduce the duration of the post-processing phase. One of the most significant disadvantages of FDM-based 3D printing is the relatively low mechanical properties of obtained products, which are often also anisotropic [38,45]. This characteristic may have contributed to the non-serious adverse events that occurred in two cases. Other additive manufacturing techniques may also be investigated: selective laser sintering (SLS) is known to improve material isotropy and mechanical properties [17], whereas robotic arm printing may allow for the production of 3D structures on curved surfaces with fewer supports [46]. In respect to other works presenting patterns of holes and openings [26], we had to face the issue of retraction with PETG with subsequent bubbling. Those patterns are easily obtainable with other more expensive 3D printing technologies (e.g., laser sintering or multi-jet fusion) or investigating other materials and print setups for FDM (e.g., reducing speed, but losing time efficiency).

### 4.4. Post-Processing

PETG material was suitable for post-processing, even though greater caution is required in drilling and manual milling compared with that of a conventional brace, to avoid the generation of cracks that may lead to brace failure. No previous works have discussed the post-print machinability of PETG in the orthopedic field.

### 4.5. Postural Stability

This is the first study comparing the effects of the three conditions, namely unbraced, braced with a conventional orthosis, and braced with a 3D-printed orthosis, using standard validated sway metrics collected with a wearable inertial sensor [41,42]. The results are promising because we did not find any statistically significant differences between the two braced conditions, meaning that the additive manufacturing process is suitable to produce braces with at least similar effects on postural stability compared with conventional orthoses. We would like to emphasize that we do not claim improved posture of the patients in our study from the use of 3D-printed orthoses. However, we found statistically significant improvements in standing balance parameters between braced and unbraced conditions. This finding confirms what was suggested by Paolucci and colleagues [31]. It is worth noting that the 3D-printed brace improved time-domain parameters such as sway range and root mean square, whereas the conventional brace had a positive effect on frequency-domain parameters. Further studies should clarify if and to what extent the 3D-printed braces influence standing balance and its underlying mechanisms. 

### 4.6. Limitations

In addition to the improvements that can be made to the production process discussed above, the present study has some limitations. First, the sample size of this study was small (n = 10), such that the findings lack generalizability and were confined to a preliminary exploratory study. In addition, the braces were worn for only a short period of time; longer studies will be needed to evaluate the clinical effectiveness of these additive manufactured braces, including long-term comfort and durability.

The chosen material (PETG) was suitable for producing 3D-printed back braces, but showed higher brittleness compared with the standard polypropylene used for conventional thermoformed braces, which was also observed in this study. In addition, it also evidenced minor issues for both printing and post-processing. Other materials, such as PLA, ABS, and nylon, can be used for orthosis production [9], and should be further investigated. Although we acknowledge that additive manufactured parts have inferior strength compared with bulk material parts, FDM remains to be a promising technique for scoliosis brace production.

An additional limitation of the present work was the limited quantitative comparisons between printed and thermoformed braces, restricted to the assessment of patient postural stability. Questionnaires investigating acceptance, safety, and satisfaction of the printed braces in comparison with conventional products may provide further insights. Finally, future research should include measures such as digital image correlation, which may provide useful quantitative indications on the geometrical deviations of the braces obtained by additive manufacturing and those produced using conventional methods.

## 5. Conclusions

Spinal orthoses produced by an innovative production process based on digital scans, CAD, and FDM 3D printing are valid alternatives to conventionally produced orthoses. Additive manufacturing proved to be the most critical phase in the whole process, due to the intrinsic limits of FDM methodology and significant number of parameters requiring fine-tuning for optimal results, whereas 3D scanning, CAD design, and post-processing did not pose significant challenges. There is room for improvement to reduce material waste and time requirements. The tested spinal braces provided equivalent postural stability compared with conventional orthoses in patients with scoliosis.

## Figures and Tables

**Table 1:** Three-dimensional printing parameters. Ranges are provided where applicable. NA: not applicable.

Parameter	Typical Value	Range
Layer height	0.6 mm with a 1.2 mm nozzle	0.4–0.6 mm
Line width	1.1 mm	1.0–1.25 mm
Wall thickness	2.2 mm	2.0–2.5 mm
Z-seam alignment	Close to the brace aperture	NA
Infill density	No infill (only wall lines)	NA
Printing temperature	240 °C	230–250 °C
Build plate temperature	90 °C	NA
Flow	110%	103–110%
Speed	35 mm/s	25–35 mm/s
Build plate adhesion type	Brim (5 lines)—150 mm	NA

**Table 2:** Criteria for the evaluation of the four production phases of the 3D-printed braces.

Production Phase	Level	Criteria Description
3D chest acquisition	Not acceptable	More than five attempts were needed for a successful scan
	Acceptable	Four or five attempts were needed for a successful scan
	Good	Two or three attempts were needed for a successful scan
	Optimal	The scan was performed successfully at the first attempt
CAD design	Not acceptable	The CAD design was successful after more than 3 h
	Acceptable	The CAD design process lasted 2 to 3 h
	Good	The CAD design process lasted 1 to 2 h
	Optimal	The CAD design process lasted less than 1 h
Additive manufacturing	Not acceptable	Production was successful after more than three attempts
	Acceptable	Production was successful at the third attempt
	Good	Production was successful at the second attempt
	Optimal	Production was successful at the first attempt
Post-processing	Not acceptable	Post-processing lasted more than 1.5 h
	Acceptable	Post-processing lasted 1 to 1.5 h
	Good	Post-processing lasted 0.5 to 1 h
	Optimal	Post-processing lasted less than 0.5 h

**Table 3:** Postural stability metrics computed from accelerometry signals collected at the waist.

Balance Metric	Definition of Metric
Range	Range in the mediolateral (mL) and anteroposterior (ap) directions (m/s^2^)
Root mean square	Acceleration root mean square in the mediolateral (mL) and anteroposterior (ap) directions (m/s^2^)
Sway path length	Accelerometer trajectory length in the horizontal plane (m/s^2^)
95% Ellipse sway normalized area	Elliptical area that encapsulates 95% of the accelerometer sway path in the horizontal plane, normalized to the duration of the test (m^2^/s^5^)
Normalized jerk index	First time derivative of the acceleration signal, normalized to the duration of the test (-)
Frequency dispersion	Measure of the variability of the frequency content (occupied bandwidth) of the power spectral density, in the mediolateral and anteroposterior directions, zero for pure sinusoid, increases with spectral bandwidth to one (-)

**Table 4:** Evaluation of the production process phases for each 3D-printed orthosis. # = Number of total attempts).

ID	3D Chest Acquisition	CAD Design Process	Additive Manufacturing	Post-Processing
1	Optimal (#1)	Not acceptable (1 h 40 min)	Not acceptable (#5)	Acceptable (1 h 40 min)
2	Optimal (#1)	Good (1 h 40 min)	Optimal (#1)	Good (40 min)
3	Optimal (#1)	Acceptable (2 h)	Good (#2)	Good (45 min)
4	Optimal (#1)	Good (1 h 40 min)	Optimal (#1)	Acceptable (1 h 20 min)
5	Acceptable (#5)	Good (1 h)	Optimal (#1)	Acceptable (1 h 40 min)
6	Optimal (#1)	Good (1 h 20 min)	Optimal (#1)	Acceptable (1 h 20 min)
7	Optimal (#1)	Good (1 h 20 min)	Optimal (#1)	Acceptable (1 h 20 min)
8	Optimal (#1)	Good (1 h 20 min)	Optimal (#1)	Good (1 h)
9	Optimal (#1)	Good (1 h 20 min)	Optimal (#1)	Good (50 min)
10	Optimal (#1)	Optimal (40 min)	Optimal (#1)	Good (1 h)

## Data Availability

The data presented in this study are openly available in Zenodo at (https://doi.org/10.5281/zenodo.6922447, accessed on 5 September 2022).
